# The additive effects of atorvastatin and insulin on renal function and renal organic anion transporter 3 function in diabetic rats

**DOI:** 10.1038/s41598-017-13206-5

**Published:** 2017-10-19

**Authors:** Laongdao Thongnak, Anchalee Pongchaidecha, Krit Jaikumkao, Varanuj Chatsudthipong, Nipon Chattipakorn, Anusorn Lungkaphin

**Affiliations:** 1 Department of Physiology, Faculty of Medicine, Chiang Mai University, Chiang Mai, Thailand; 2 Department of Physiology, Faculty of Science, Mahidol University, Bangkok, Thailand; 3 Cardiac Electrophysiology Research and Training Center, Faculty of Medicine, Chiang Mai University, Chiang Mai, Thailand

## Abstract

Hyperglycemia-induced oxidative stress is usually found in diabetic condition. 3-hydroxy-3-methylglutaryl coenzyme-A (HMG-CoA) reductase inhibitors, statins, are widely used as cholesterol-lowering medication with several “pleiotropic” effects in diabetic patients. This study aims to evaluate whether the protective effects of atorvastatin and insulin on renal function and renal organic anion transporter 3 (Oat3) function involve the modulation of oxidative stress and pancreatic function in type 1 diabetic rats. Type 1 diabetes was induced by intraperitoneal injection of streptozotocin (50 mg/kg BW). Atorvastatin and insulin as single or combined treatment were given for 4 weeks after diabetic condition had been confirmed. Diabetic rats demonstrated renal function and renal Oat3 function impairment with an increased MDA level and decreased SOD protein expression concomitant with stimulation of renal Nrf2 and HO-1 protein expression. Insulin plus atorvastatin (combined) treatment effectively restored renal function as well as renal Oat3 function which correlated with the decrease in hyperglycemia and oxidative stress. Moreover, pancreatic inflammation and apoptosis in diabetic rats were ameliorated by the combined drugs treatment. Therefore, atorvastatin plus insulin seems to exert the additive effect in improving renal functionby alleviating hyperglycemiaand the modulation of oxidative stress, inflammation and apoptosis.

## Introduction

Type 1 diabetes (T1D) is an autoimmune disease characterized by low plasma insulin due to a destruction of the pancreatic β-cells which synthesize insulin^1^ leading to the development of hyperglycemia. Diabetic nephropathy (DN) is a devastating complication of type 1 diabetes, which is the leading cause of end-stage renal disease (ESRD) and a major cause of morbidity and mortality in T1D patients^2^. Although the pathogenesis of DN is still not fully understood it has been suggested that long-term hyperglycemia activates reactive oxygen species (ROS) production by increasing advanced glycation end products (AGE). Subsequently, AGE activates the polyol pathway resulting in the activation of Nicotinamide adenine dinucleotide phosphate (NADPH) oxidase causing cell damage and dysfunction^3–5^. These conditions can lead to diabetic nephropathy and diabetic-induced complications in several organs including the pancreas. DN is characterized by various ultrastructural changes of nephrons including basement membrane thickening, glomerular and tubular hypertrophy, glomerulosclerosis and tubulointerstitial fibrosis^6^. This pathology markedly affects the secretory and excretory capacities of transporters in renal proximal tubules.

The organic anion transporter 3 (Oat3) is an important renal transporter which is localized in the basolateral membrane of the renal proximal tubule. It plays an essential role in renal excretion of a variety of drug metabolites, endogenous substances, and environmental toxins. Our previous study has demonstrated that a decrease in function and expression of renal Oat3 in diabetic rats were associated with an increased oxidative stress level from hyperglycemia^7^. Moreover, we found that an impairment of renal Oat3 transport function and expression in diabetic rats was restored by insulin treatment^7,8^.

Atorvastatin, an 3-hydroxy-3-methylglutaryl coenzyme-A (HMG-CoA) reductase inhibitor, is widely used to treat hypercholesterolemia and dyslipidemia in diabetic patients. A recent study demonstrated the pleiotropic effects of statins in attenuating oxidative stress, inflammation, apoptosis and thrombosis^9^. In addition, cardioprotective effects of statins in an angiotensin II (Ang II)-induced cardiac hypertrophy and fibrosis mice model^10^ and the renoprotective effects of statins in gentamicin-induced nephropathy in rats through the attenuation of oxidative stress leading to improving renal Oat3 and renal function^11^ have been reported by our team. We also demonstrated that renal inflammation, endoplasmic reticulum (ES) stress and apoptosis were ameliorated by atorvastatin in gentamicin-induced nephrotoxicity in rats^12^. In contrast-induced nephropathy, rosuvastatin was found to modulate nitric oxide synthesis, inflammation, oxidative stress and apoptosis in diabetic male rats^13^. Taken together, either insulin or atorvastatin can improve renal function in diabetic rats but their combined effect has not been investigated. In addition, we were very interested in the effect of the combined treatment on pancreatic function and whether it was effective in the modulation of insulin secretion in diabetic condition. Therefore, in this study we have evaluated the renoprotective effects of atorvastatin plus low dose insulin treatment on renal function and the function of the important renal transport protein, renal Oat3, in modulation of the renal oxidative stress pathway, and its effect on the inflammation and apoptosis of the pancreas in streptozotocin (STZ)-induced diabetic rats.

## Results

### Effects of pharmacological intervention on metabolic parameters in STZ-induced diabetic rats

As shown in Table 1, type 1 diabetic rats showed a significant decrease in body weight and plasma insulin level when compared with those of the control and control plus atorvastatin rats (p < 0.05). Plasma glucose, cholesterol, triglyceride, urine glucose and urine volume were significantly increased in diabetic rats when compared with control or control plus atorvastatin-treated rats (p < 0.05). Treatment with insulin as a single entity or combined with atorvastatin correlated with significantly increased body weight compared with diabetic rats (p < 0.05). Similarly, the rats that received the combined drug treatment (atorvastatin plus insulin) had significantly higher body weight than those receiving only a single treatment of either insulin or atorvastatin-treated rats (p < 0.05). Rats on insulin or atorvastatin treatment alone and combined drugs treatment showed significantly decreased fasting blood glucose levels when compared with those of diabetic rats (p < 0.05). However, the fasting blood glucose in the combined drug treatment was lowest and significantly different from that of insulin or atorvastatin-treated rats (p < 0.05). Co-administration of insulin and atorvastatin had a marked effect on the restoration of plasma insulin which nearly returned to normal levels and was significantly different when compared to that of diabetic, insulin or atorvastatin-treated rats (p < 0.05). Also, rats having the combined drug treatment showed a significantly lower plasma cholesterol level when compared to diabetic and atorvastatin-treated rats (p < 0.05). In addition, rats receiving insulin or atorvastatin alone or the combined therapy showed significantly lowered urine glucose concentration when compared to that of diabetic rats (p < 0.05). Rats having insulin as a single or combined treatment with atorvastatin showed a significantly decreased 24-hr urine volume in comparison with that of diabetic rats (p < 0.05). Surprisingly, atorvastatin treatment significantly increased 24-hr urine volume with respect to diabetic, insulin-treated and combined-treated rats (p < 0.05).

**Table 1:** Effects of pharmacological interventions on metabolic parameters.

	Control	DM	DMI	DMS	DMIS	CS
Body weight (g)	396.67 ± 17.21	245 ± 14.50*	302.5 ± 17.69*^†#^	219 ± 6.11*	344.17 ± 14.17*^†‡#^	400 ± 12.18^†‡#^
Fasting blood glucose (mg/dl)	154.85 ± 13.93	565.57 ± 37.03*	467.04 ± 40.30*^†^	422.94 ± 31.92*^†^	254.92 ± 32.82*^†‡#^	131.08 ± 5.45^†‡#^
Plasma insulin (U/ml)	2.47 ± 0.43	0.57 ± 0.24*	0.85 ± 0.19*	0.65 ± 0.20*	2.20 ± 0.71^†‡#^	3.23 ± 0.48^†‡#^
Plasma cholesterol (mg/dl)	74.71 ± 3.99	102.92 ± 5.97*	91.88 ± 1.55*	95.01 ± 4.89*	81.13 ± 1.67^†#^	70.87 ± 4.62^†‡#^
Plasma triglyceride (mg/dl)	75.27 ± 6.98	166.65 ± 28.62*	163.18 ± 16.23*	154.62 ± 26.57*	122.22 ± 12.35	78.51 ± 8.09^†‡#^
Urine glucose (mg/dl)	115.84 ± 5.84	8834.1 ± 375.5*	6350.6 ± 831.9*^†^	6136.9 ± 802.7*^†^	6056.9 ± 426.5*^†^	110.41 ± 3.08^†‡#^
Urine volume (ml/24 h.)	18.83 ± 0.83	216.33 ± 14.70*	133.67 ± 15.75*^†#^	251.67 ± 6.60*^†^	135.50 ± 8.43*^†#^	18.67 ± 1.61^†‡#^

Data presented are means ± SEM. n = 6 rats per group. C - control group; DM - diabetic group; DMI - diabetic plus insulin group; DMS - diabetic plus atorvastatin group; DMIS - diabetic and insulin plus atorvastatin group; CS - control plus atorvastatin group. *p < 0.05 vs. control and control plus atorvastatin groups, ^†^p < 0.05 vs diabetic group, ^‡^p < 0.05 vs. diabetic plus insulin group, ^*#*^p < 0.05 vs. diabetic plus atorvastatin group.

### Effects of pharmacological intervention on kidney weight, kidney weight to body weight ratio, and renal function in STZ-induced diabetic rats

Type 1 diabetic rats showed significant increases in kidney weight (KW) and kidney weight to body weight (KW/BW) ratio compared with the control and control plus atorvastatin rats (p < 0.05) (Table 2). Although, the KW/BW ratio of both insulin and combined drug treatment rats (p < 0.05) was significantly lower than that of diabetic rats. The greater reduction in KW/BW ratio was observed in the combined drug treatment (insulin plus atorvastatin) group (p < 0.05).

**Table 2:** Effects of pharmacological interventions on renal functions.

	**Control**	**DM**	**DMI**	**DMS**	**DMIS**	**CS**
Kidney weight (g)	1.10 ± 0.02	1.34 ± 0.06*	1.38 ± 0.06*	1.25 ± 0.03*	1.29 ± 0.04*	1.05 ± 0.03^†‡#^
Kidney weight /Body weight ratio(10^−3^)	2.79 ± 0.01	5.55 ± 0.38*	4.66 ± 0.36*^†#^	5.81 ± 0.28*	3.79 ± 0.21*^†‡#^	2.64 ± 0.10^†‡#^
BUN (mg/dl/g BW)	0.05 ± 0.01	0.22 ± 0.03*	0.10 ± 0.01*^†#^	0.19 ± 0.01*	0.06 ± 0.01^†#^	0.05 ± 0.01^†‡#^
Creatinine (10^−3^mg/dl/g BW)	1.15 ± 0.04	2.13 ± 0.15*	1.82 ± 0.22*	1.74 ± 0.12*^†^	1.08 ± 0.06^†‡#^	0.99 ± 0.07^†‡#^
Urine creatinine (mg/dl)	56.64 ± 7.42	6.37 ± 0.92*	14.45 ± 3.95*	5.88 ± 0.57*	29.36 ± 9.61*^†#^	54.57 ± 7.59^†‡#^
eGFR (% of control)	100 ± 1.29	52.69 ± 4.43*	67.11 ± 2.67*^†#^	50.58 ± 1.41*	75.77 ± 3.30*^†‡#^	92.31 ± 2.81^†‡#^

Data presented are means ± SEM. n = 6 rats per group. C - control group; DM - diabetic group; DMI - diabetic plus insulin group; DMS - diabetic plus atorvastatin group; DMIS - diabetic and insulin plus atorvastatin group; CS - control plus atorvastatin group. eGFR, estimated glomerular filtration rate calculated as follow:- eGFR = (urine creatinine x urine flow rate)/serum creatinine. *p < 0.05 vs. control and control plus atorvastatin groups, ^†^p < 0.05 vs. diabetic group, ^‡^p < 0.05 vs. diabetic plus insulin group, ^#^p < 0.05 vs. diabetic plus atorvastatin group.

Significant increases in serum blood urea nitrogen (BUN) and creatinine levels, along with a decrease in urine creatinine level and estimated glomerular filtration rate (eGFR) in diabetic rats, compared with that of the control or control plus atorvastatin rats (p < 0.05) were observed which indicated an impairment in renal function in rats with the diabetic condition (Table 2). The greatest reduction in BUN was found in the combined drug treatment group. Of note, the decreased serum BUN and creatinine levels, in this group showed a strong correlation with an improved eGFR. The results suggested that combined drug treatment produced the greatest effect in improving kidney function in the diabetic rats.

### Effects of atorvastatin on kidney histological change in STZ-induced diabetic rats

**Figure 1:**
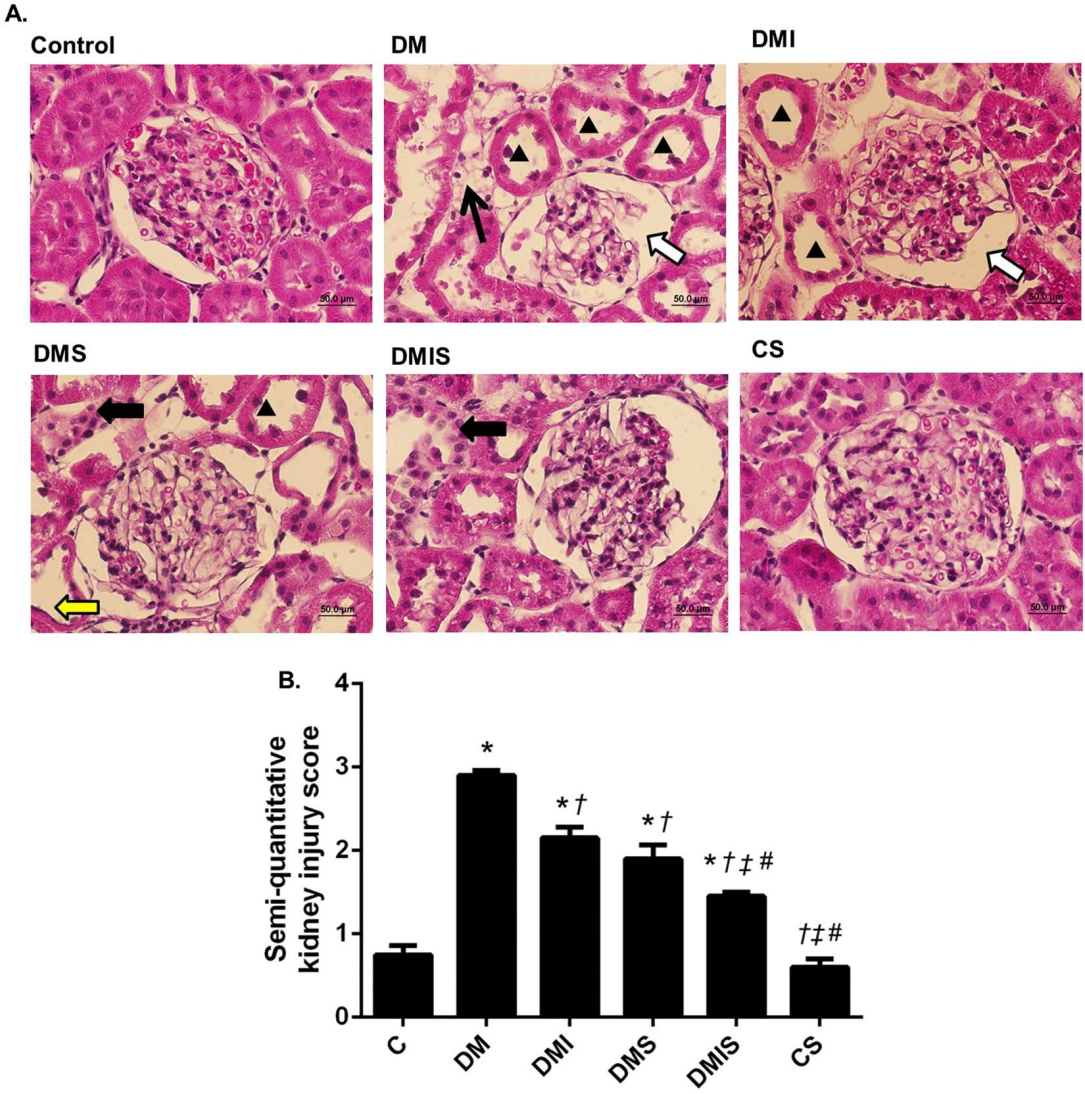
(**A**) Photomicrographs of histological sections of kidney stained with hematoxylin and eosin (H&E) (x40); images of glomeruli and renal tubules from control, diabetic (DM), diabetic plus insulin (DMI), diabetic plus atorvastatin (DMS), diabetic and insulin plus atorvastatin (DMIS) groups, and control plus atorvastatin (CS) are indicated. (▲) Shows dilatation of renal tubular; (arrow) represents interstitial fibrosis; (white arrow) represents capsular space of the glomerular capsule; (black arrow) represents neutrophil accumulation; (yellow arrow) represents tubular atrophy. (**B**) Quantitative analysis of diabetic injury kidney was determined by semi-quantitative kidney injury scoring (0–4); Bar graphs presented show mean ± SEM. n = 5 rats per group. C - control group; DM - diabetic group; DMI - diabetic plus insulin group; DMIS - diabetic and insulin plus atorvastatin group; DMS - diabetic plus atorvastatin group; CS - control plus atorvastatin. *p < 0.05 vs. control group and control plus atorvastatin groups, ^†^p < 0.05 vs. diabetic group, ^‡^p < 0.05 vs. diabetic plus insulin group, and ^#^p < 0.05 vs. diabetic plus atorvastatin group.

As shown in Fig. 1A, moderate glomerular lesions were found in diabetic rats when compared with control and control plus atorvastatin rats. Glomerular capillaries were attached to the Bowman’s capsule, their wide and irregular morphology leading to increased capsule space in the diabetic group. In the kidneys from the diabetic rats, tubulointerstitial damage was found with epithelial cell atrophy. Furthermore, a small amount of focal interstitial fibrosis with neutrophil accumulation was observed. These results were supported by the significant increase in renal injury score when compared with control (p < 0.05) (Fig. 1B). Atorvastatin plus insulin (combined) treatment led to a decrease in these morphological changes as indicated by a significant improve in renal injury score when compared with diabetic group (p < 0.05). Moreover, the combined treatment found to have a significant different in renal injury score when compared to insulin or atorvastatin treatment alone (p < 0.05).

### Effects of pharmacological intervention on renal Oat3 function and expression in STZ-induced diabetic rats

**Figure 2:**
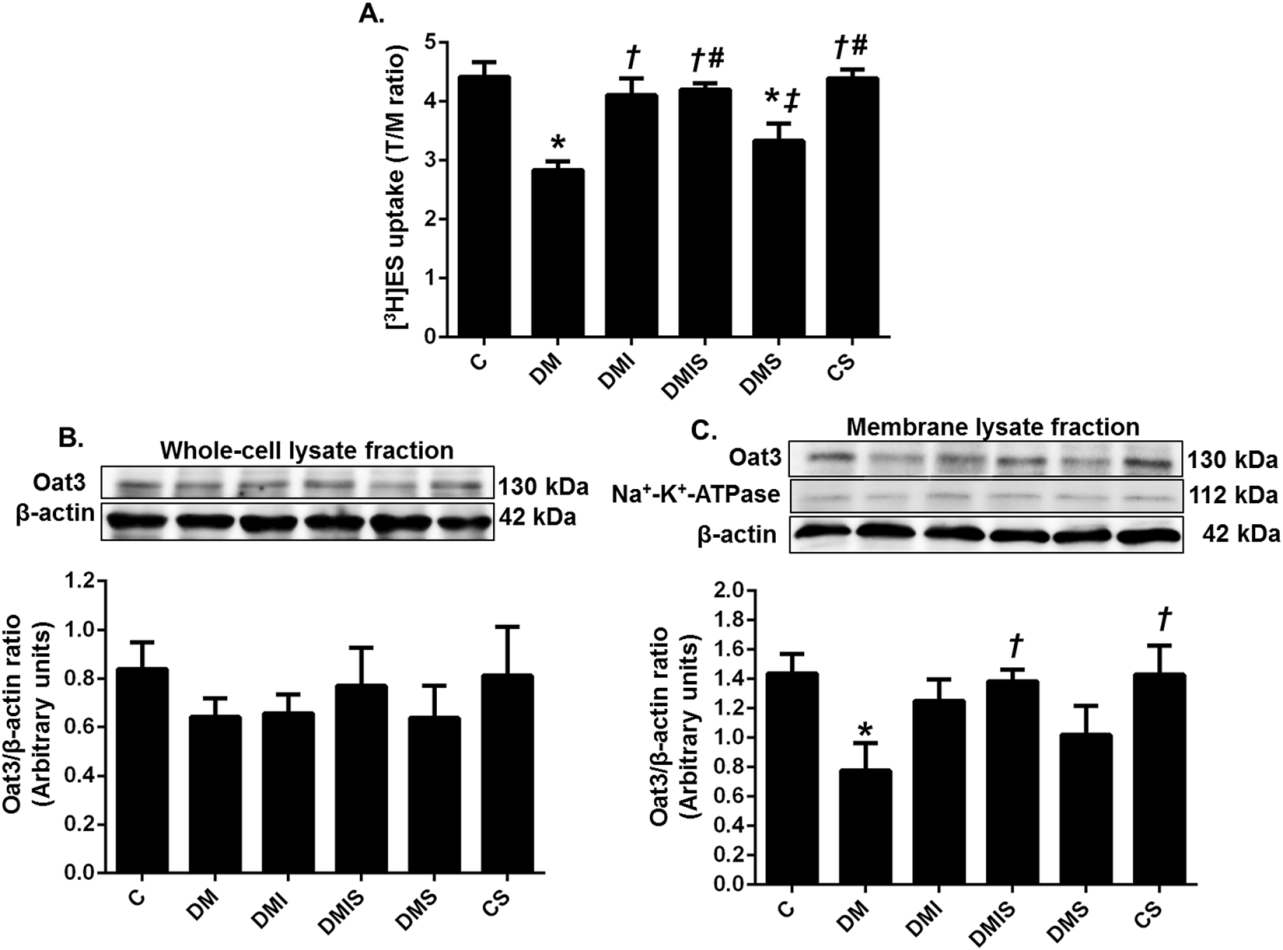
Effects of pharmacological intervention on renal cortical Oat3 function and expression. [^3^H]ES uptake calculated from tissue/medium ratio. (**A**) Western blot analysis of Oat3 expression in whole cell lysate fraction normalized by β-actin (**B**) and in membrane lysate fraction (cropped blots) normalized by β-actin (**C**). Na^+^-K^+^ ATPase was used as a marker for the membrane fraction. Full-length blots are presented in Supplementary Figure 1. Bar graphs presented show mean ± SEM. n = 6 rats per group. C - control group; DM - diabetic group; DMI - diabetic plus insulin group; DMIS - diabetic and insulin plus atorvastatin group; DMS - diabetic plus atorvastatin group; CS - control plus atorvastatin. *p < 0.05 vs. control group and control plus atorvastatin groups, ^†^p < 0.05 vs. diabetic group, ^‡^p < 0.05 vs. diabetic plus insulin group, and ^#^p < 0.05 vs. diabetic plus atorvastatin group.

An impairment in renal Oat3 function in diabetic rats was indicated by a significant decrease in [^3^H]estrone sulfate (ES) uptake when compared with the control or control plus atorvastatin rats (p < 0.05) (Fig. 2A). Insulin as a single or combined treatment with atorvastatin led to significantly elevated [^3^H]ES uptake in comparison with diabetic rats (p < 0.05). Although, atorvastatin treatment led to a tendency to increase [^3^H]ES uptake it was still significantly lower than that of the control group. As presented in Fig. 2B,C, the expression of renal Oat3 in the whole cell lysate of cortical tissues was comparable among the experimental groups while renal Oat3 expression in the membrane fraction was significantly decreased in diabetic rats (p < 0.05). All drug treatment groups indicated a tendency to increase [^3^H]ES uptake. However, a significant increase in renal Oat3 expression in the membrane fraction was only observed in the combined drug treatment rats (p < 0.05) but there was an increasing trend noted in insulin-treated rats. These findings indicated that the decreased Oat3 function associated with the down-regulation of Oat3 in the membrane of renal tubular cells in cases of diabetes mellitus may well be improved by insulin or insulin plus atorvastatin treatment.

### Effects of pharmacological intervention on oxidative stress status in STZ-induced diabetic rats

**Figure 3:**
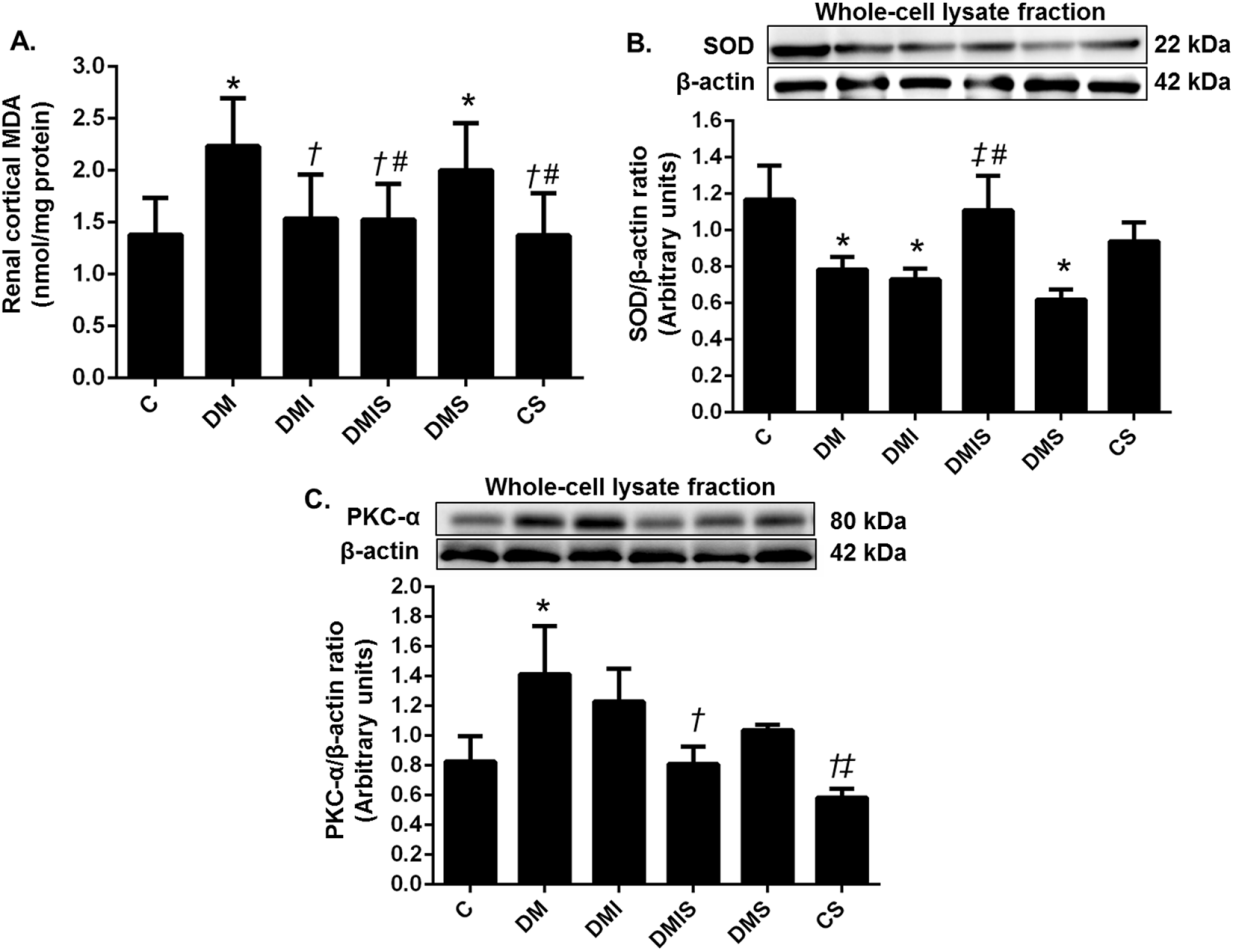
Effects of pharmacological intervention on renal cortical MDA level (**A**), renal cortical expression of SOD (**B**) and PKC-α (**C**). Western blot analysis of SOD and PKC-α expression in the whole cell lysate fraction of renal cortical tissues (cropped blots) normalized by β-actin. Full-length blots are presented in Supplementary Figure 2. Bar graphs presented show mean ± SEM. n = 6 rats per group. C - control group; DM - diabetic group; DMI - diabetic plus insulin group; DMIS - diabetic plus insulin and atorvastatin group; DMS - diabetic plus atorvastatin group; CS - control plus atorvastatin. *p < 0.05 vs. control group and control plus atorvastatin groups, ^†^p < 0.05 vs. diabetic group, ^‡^p < 0.05 vs. diabetic plus insulin group, and ^#^p < 0.05 vs. diabetic plus atorvastatin group.

As shown in Fig. 3, significant increases in malondialdehyde (MDA) level and protein kinase C alpha (PKC-α) expression along with an apparent decrease in superoxide dismutase (SOD) expression were observed in the renal cortical tissues of diabetic rats compared with that of the control rats (p < 0.05) reflecting an increase in oxidative stress in diabetes. Insulin as a single or combined treatment with atorvastatin led to a significantly decreased renal cortical MDA level when compared with diabetic rats (p < 0.05) and this value was approaching the control level. The atorvastatin treatment alone seemed to have no effect on cortical MDA. Interestingly, diabetic rats on the combined drug treatment showed a significant increase in the expression of SOD (p < 0.05) with compared to diabetic rats and the ones with insulin or atorvastatin treatment alone. Moreover, rats on the combined drug treatment showed a significant decrease in the expression of PKC-α when compared with the diabetic rats (p < 0.05). The results from this study suggested that atorvastatin plus insulin treatment could markedly decrease oxidative stress in the diabetic condition.

### Effects of pharmacological intervention on the oxidative stress pathway in STZ-induced diabetic rats

**Figure 4:**
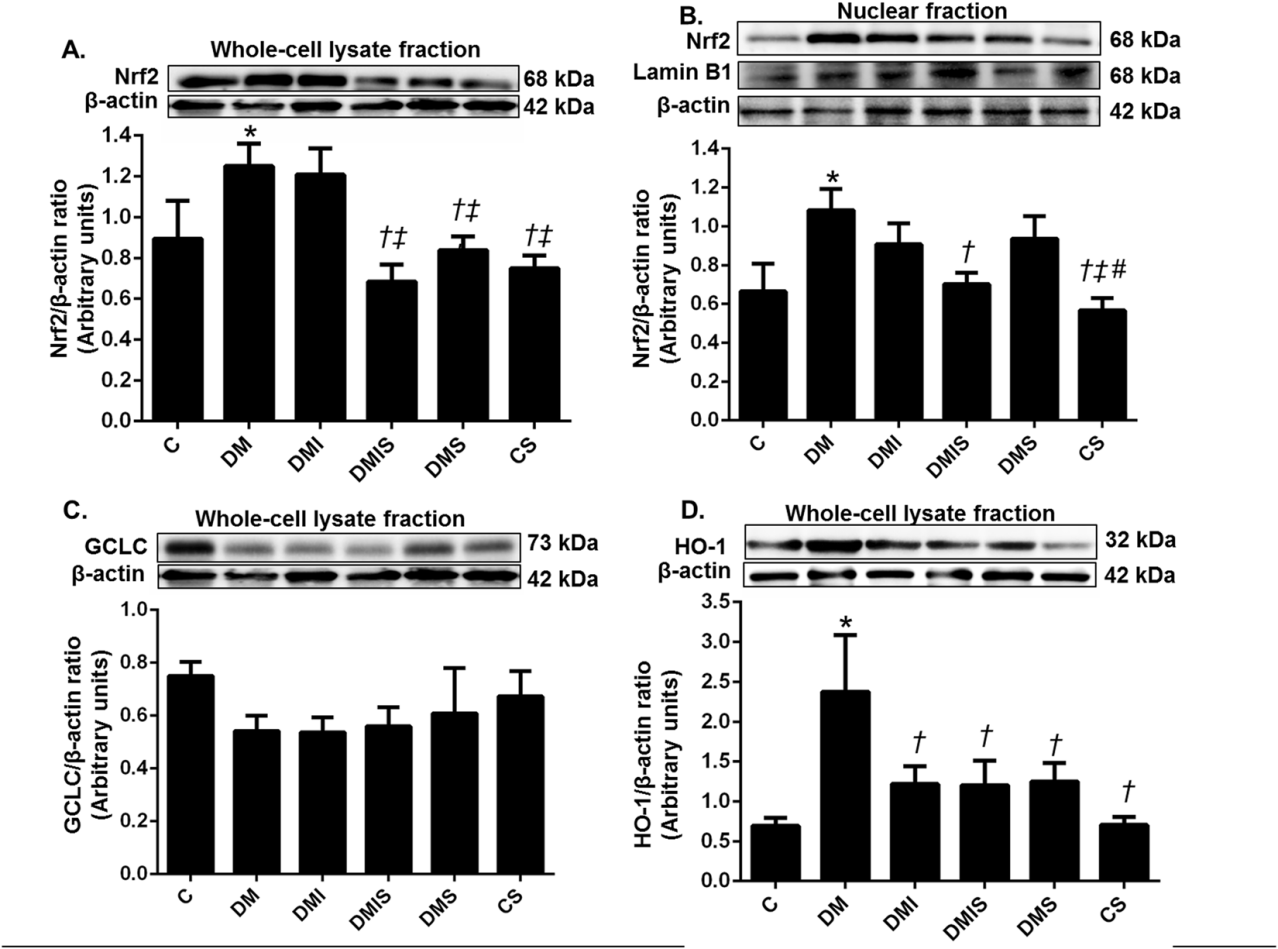
Effects of pharmacological intervention on the renal cortical expression of Nrf-2, GCLC and HO-1. Western blot analysis of Nrf2 expression in nuclear (**A**), Nrf2 in whole cell lysate fractions (**B**), GCLC (**C**), and HO-1 (**D**) of renal cortical tissues (whole cell lysate fraction) (cropped blots) normalized to β-actin. Lamin B1 was used as a marker for the nuclear fraction. Full-length blots are presented in Supplementary Figure 3. Bar graphs presented show mean ± SEM. n = 6 rats per group. C - control group; DM - diabetic group; DMI - diabetic plus insulin group; DMIS - diabetic and insulin plus atorvastatin group; DMS - diabetic plus atorvastatin group; CS - control plus atorvastatin. *p < 0.05 vs. control group and control plus atorvastatin groups, ^†^p < 0.05 vs. diabetic group, ^‡^p < 0.05 vs. diabetic plus insulin group, and ^*#*^p < 0.05 vs. diabetic plus atorvastatin group.

To further study the mechanism by which combined drug treatment led to decreased hyperglycemia-induced oxidative stress, the expression of proteins involved in the oxidative stress pathway, including nuclear factor erythroid 2-related factor 2 (Nrf2), glutamate-cysteine ligase catalytic subunit (GCLC), and heme oxygenase-1 (HO-1) were investigated in the renal cortical tissues. Diabetic rats showed a significant increase in the expression of Nrf2 in both the nuclear and whole cell lysate fractions of renal cortical tissues compared with the control and control plus atorvastatin rats (p < 0.05) (Fig. 4). These results indicated, not only was there an increase in Nfr2 protein synthesis in the cell, but there was also increased translocation of Nrf2 from the cytoplasm to nucleus occurring in the diabetic rats. Interestingly, the combined drug treatment significantly reversed the expression of Nrf2 in both the whole cell lysate and the nuclear fraction when compared with that of diabetic rats (p < 0.05). Rats on atorvastatin alone had significantly attenuated Nrf2 expression in whole cell lysate fractions but there was no significant effect on Nrf2 in the nuclear fraction compared with diabetic rats (p < 0.05). Of note, Nrf2 induced a significant increase in antioxidant enzyme HO-1 in diabetic rats compared to that of control or control plus atorvastatin rats (p < 0.05) whereas there was no change of GCLC expression among the experimental groups. All types of drug treatment showed a significant lowering of the expression of HO-1 in comparison with that of the diabetic rats (p < 0.05). The results indicated that combined drug treatment inactivated Nrf2 translocation to nucleus which was related to the reduced HO-1 expression.

### Effects of pharmacological intervention on the pancreatic apoptotic pathway in STZ-induced diabetic rats

**Figure 5:**
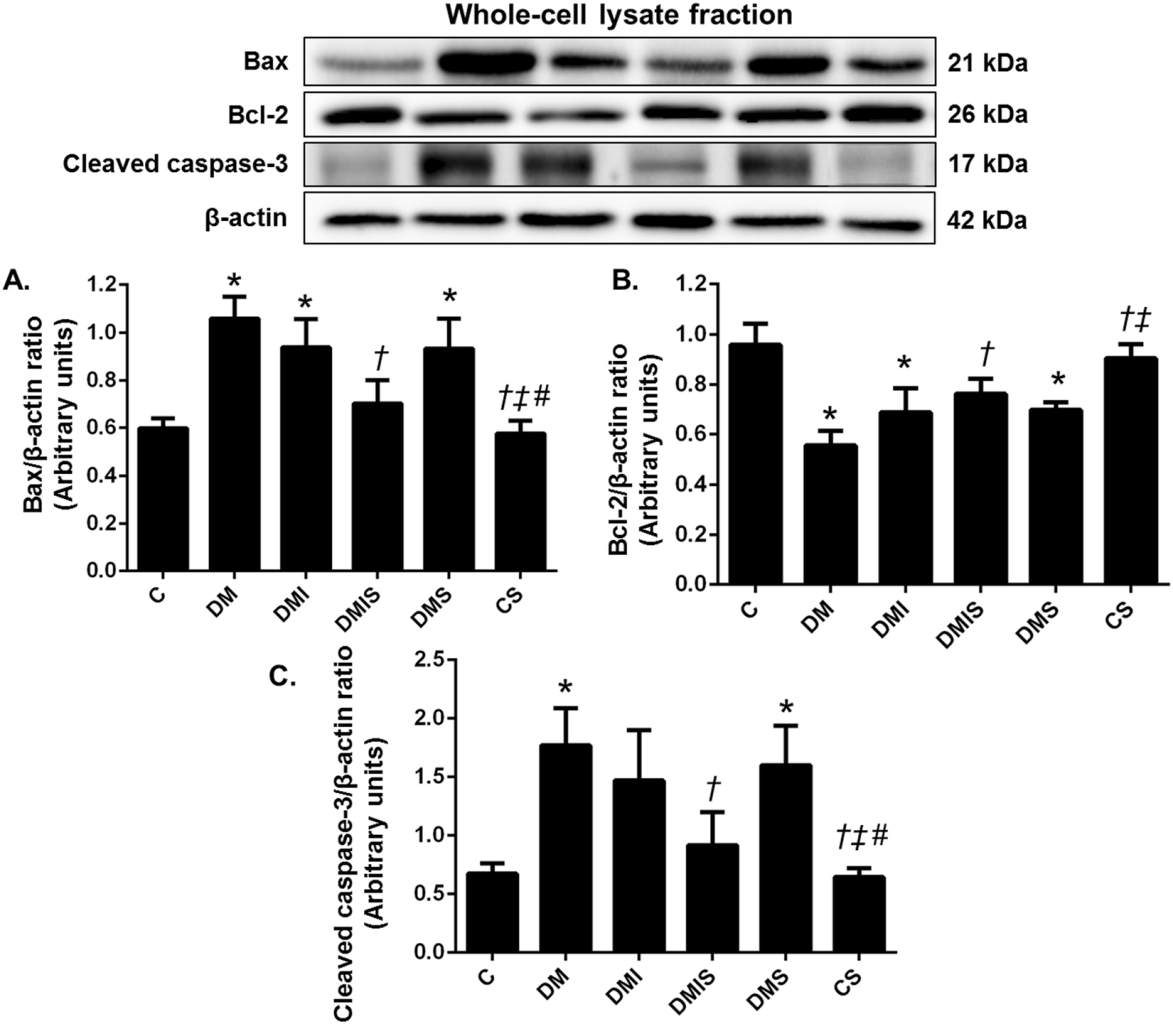
Effects of pharmacological intervention on the expression of Bax, Bcl-2, and cleaved caspase-3 in pancreatic tissues. Western blot analysis showing the expressions of Bax (**A**), Bcl-2 (**B**), and cleaved caspase-3 (**C**) in pancreatic tissues (cropped blots) normalized to β-actin. Full-length blots are presented in Supplementary Figure 4. Bar graphs presented show mean ± SEM. n = 6 rats per group. C - control group; DM - diabetic group; DMI - diabetic plus insulin group; DMIS - diabetic and insulin plus atorvastatin group; DMS - diabetic plus atorvastatin group; CS - control plus atorvastatin. *p < 0.05 vs. control group and control plus atorvastatin groups, ^†^p < 0.05 vs. diabetic group, ^‡^p < 0.05 vs. diabetic plus insulin group, and ^#^p < 0.05 vs. diabetic plus atorvastatin group.

Atorvastatin has been shown to inhibit the apoptosis of myocardial cells in cases of heart failure following myocardial infarction in rats^14^. As plasma insulin levels were found to decrease in diabetic type 1 rats and the combined drug treatment led to the restoration of these levels to within normal limits, it is likely that pancreas function recovered after the treatment. To verify this hypothesis the expression of apoptotic proteins in pancreatic tissue was investigated. As shown in Fig. 5, diabetic rats showed a significant increase in B-cell lymphoma 2 (Bcl-2) associated X (Bax) protein and cleaved caspase-3 expression, and marked reduction of Bcl-2 expression in pancreatic tissue when compared with that of the control or control plus atorvastatin rats (p < 0.05). The combined drug treatment led to a significant reduction of Bax and cleaved caspase-3 expression and apparently enhanced the expression of Bcl-2 in comparison with diabetic rats (p < 0.05) whereas insulin or atorvastatin treatment alone had no effects on these parameters. These results suggested that combined drug treatment protected against pancreatic apoptosis which helps preserve the pancreas tissue and function in diabetic rats.

### Effects of pharmacological intervention on pancreatic inflammation pathways in STZ-induced diabetic rats

**Figure 6:**
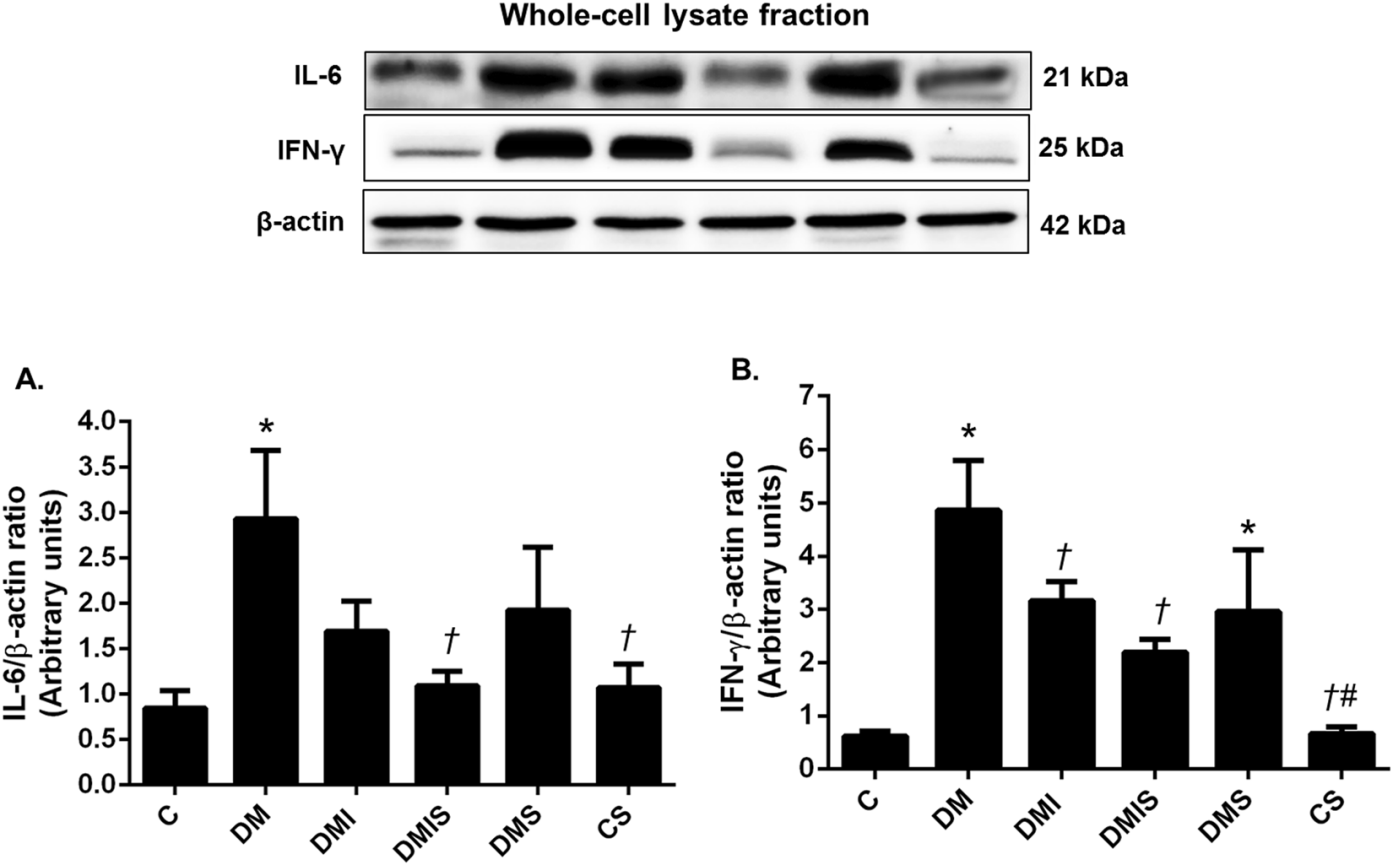
Effects of pharmacological intervention on the expression of IL-6, and IFN-γ in pancreatic tissues. Western blot analysis showing the expression of IL-6 (**A**), and IFN-γ (**B**) in pancreatic tissues (cropped blots) normalized to β-actin. Full-length blots are presented in Supplementary Figure 5. Bar graphs presented show mean ± SEM. n = 6 rats per group. C - control group; DM - diabetic group; DMI - diabetic plus insulin group; DMIS - diabetic and insulin plus atorvastatin group; DMS - diabetic plus atorvastatin group; CS - control plus atorvastatin. *p < 0.05 vs. control group and control plus atorvastatin groups, ^†^p < 0.05 vs. diabetic group, ^‡^p < 0.05 vs. diabetic plus insulin group, and ^#^p < 0.05 vs. diabetic plus atorvastatin group.

It has been reported that chronic hyperglycemia can cause β-cell degranulation and reduction^15^. It has been shown that statins have anti-inflammatory effects in asymmetrical dimethylarginine-induced inflammatory endothelial cells^16^. We, therefore, went on to investigate the effect of atorvastatin treatment on the inflammation of pancreatic tissues. As shown in Fig. 6, there was significantly increased interferon gamma (IFN-γ) and interleukin-6 (IL-6) protein expression in diabetic rats when compared with that of the control and control plus atorvastatin rats (p < 0.05). The single treatment with either insulin or atorvastatin tended to lower levels of inflammatory proteins but the significant difference in IFN-γ was only observed in insulin-treated rats (p < 0.05) whereas the combined drug treatment led to the significant decreases seen in both IFN-γ and IL-6 protein expression when compared with diabetic rats (p < 0.05).

## Discussion

The STZ-induced diabetes type 1 in the rats in this study was characterized by body weight loss, hyperglycemia, decreased plasma insulin level, renal dysfunction and renal histological changes which were shown to have a correlation with an increase in renal oxidative stress. In addition, hyperglycemia-induced oxidative stress was found to activate the oxidative defense mechanisms through the Nrf2 pathway to protect cytotoxicity. This was consistent with findings from our previous study^7^ which reported that the increased ROS generation in diabetes activated the PKC signaling pathway resulting in the downregulation of renal Oat3 function and expression in the renal tissues. Of note, hyperglycemia also increased pancreatic expression of IL-6 and IFN-γ, indicators of inflammation, leading to pancreatic apoptosis as shown by the increase in Bax, cleaved caspase-3 protein expression and the Bax/Bcl-2 ratio. Interestingly, atorvastatin plus insulin (combined) treatment led to amelioration of the inflammation and destruction of pancreatic cells leading to an increase in insulin secretion which subsequently improved conditions of hyperglycemia. Thus, combined drug treatment could exert additive effects in preserving both renal and pancreatic functions via modulation of oxidative stress, inflammation and apoptotic pathways.

Long-term hyperglycemia leads to an increase in reactive oxygen species and reactive nitrogen species (ROS&RNS) leading to increased oxidative stress^17^. The condition of increased oxidative stress in the diabetic rats in this study was confirmed by the elevation of renal MDA, the stimulation of HO-1 expression and the reduction of antioxidant enzyme expression, SOD2. Antioxidant mechanisms are usually activated when oxidative stress occurs. The Nrf2-Kelch-like ECH-associated protein 1 (Keap1) pathway is the major regulator of cytoprotective responses and regulates the expression of antioxidant proteins which protect against oxidative damage triggered by injury and inflammation^18,19^. In the presence of ROS, cysteine residues in Keap1 are oxidized leading to a conformational change of Keap1, which prevents its binding to Nrf2. After that, Nrf2 passes by translocation into the nucleus to promote the translation of oxidative-stress-inducible genes, including HO-1^20^. In this study, an increased oxidative stress, induced by hyperglycemia in diabetic rats, led to activated Nrf2 expression and translocation, which subsequently promoted the expression of HO-1 proteins. It is now widely accepted that induction of HO-1 expression represents an adaptive response that increases cellular defense to oxidative injury. It has been reported that HO-1 is increased in livers of obese versus lean individuals and of diabetic versus nondiabetic subjects^21^. These results are supported by the previous study that found high-glucose-induced upregulation of Nrf2 and HO-1 gene expression^22^. This is in agreement with our previous reports showing the increased expression of renal Nrf2, NAD(P)H quinone dehydrogenase 1 (NQO1) and HO-1 due to oxidative stress in gentamicin-induced nephrotoxicity in rats^11,23^.

An increased expression of PKC-α in diabetic rats was also consistent with the previous report that PKC-α was activated in hyperglycemia^7^. Moreover, the decreased renal Oat3 function and expression in the present study when PKC-α was activated due to oxidative stress were similar to that observed previously in diabetic^7,8^ and gentamicin-induced nephrotoxicity in rats^11,23^.

In this study, increased pancreatic apoptosis was found to be significantly related to the decreased plasma insulin levels in diabetic rats. Hyperglycemia may negatively affect β-cell mass by inducing apoptosis without a compensatory increase in β-cell proliferation and neogenesis. It has been reported that chronic hyperglycemia can cause β-cell degranulation and reduction^15^. The increased pancreatic expression of IL-6, and IFN-γ, indicating inflammation in diabetic rats in the present study, was found to be correlated with the findings of an *in vitro* study demonstrating that the exposure of β-cells to high glucose induced interleukin 1 beta (IL-1β) which activated nuclear factor-kappa B (NF-kB) and Fas signaling and consequently triggered apoptosis^24,25^. Moreover, both IL-1β and IFN-γ also activated NF-kB and appeared to increase inducible nitric oxide synthase (iNOS) expression resulting in the stimulation of ER stress conditions and mononuclear cell activation and infiltration leading to β-cell death^26^.

Atorvastatin is a lipid-lowering agent in statins or the HMG-CoA reductase inhibitor group which inhibit the HMG-CoA reductase enzyme, the catalyst of the rate-limiting step of the mevalonate pathway^27^. It has been reported as having pleiotropic effects including anti-apoptosis^28,29^, anti-inflammation^30^, antioxidant^11,31^ and anti-thrombotic effects^32^. Although the direct effect of atorvastatin in reducing plasma levels of cholesterol was not observed in this study, the beneficial effects of the restoration of plasma insulin, reduced blood glucose, improved renal function, as well as decreased pancreatic inflammation and apoptosis were demonstrated in the diabetic rats that had undergone the combined treatment (atorvastatin with a low dose of insulin). It initiated a greater effect than that observed in the rats undergoing insulin or atorvastatin treatment alone. These pleiotropic effects of atorvastatin which led to the adjustment of glucose homeostasis and renoprotection observed in this study might be due to the additive effect of atorvastatin and insulin in controlling metabolic parameters and subsequently protect kidney dysfunction in diabetes. This proposed mechanism is supported by the previous study reporting that atorvastatin can increase β-cell function by increasing β-cell proliferation and decreasing ER stress conditions^33^. The increased insulin secretion and the attenuation of apoptosis of pancreatic tissue seen in rats on the combined treatment in this study reflected an improvement in β-cell function. The dosage of insulin used in this study had no effect on the lowering of plasma glucose to normal levels as indicated in the group on insulin treatment alone. Thus, the effective reduction of the hyperglycemic condition in the group on the combined drug treatment corroborated the pleiotropic effects of atorvastatin in the reduction of oxidative stress leading to the improvement of renal and pancreatic functions seen in this study. We found that only combined treatment had the significant effect on Nrf-2 expression while insulin, atorvastatin and combined treatment had similar effect on HO-1 expression. The results indicated that combined drug treatment inactivated Nrf2 translocation to nucleus which was related to the reduced HO-1 expression. However, the reduced HO-1 expression in insulin or atorvastatin treatment alone might be involved the other regulated mechanisms such as inflammation or insulin. Insulin has been reported to stimulate HO-1 expression in skeletal myoblast^34^. The decreased HO-1 expression might be related to the low level of plasma insulin in insulin or atorvastatin treatment alone. Thus, the combined drug treatment produced highly effective effects in the control of glucose homeostasis and the prevention of organ dysfunction when compared to of the insulin or atorvastatin treatment alone.

In conclusion, the results obtained from this study indicate that combined atorvastatin and low dose insulin treatment exhibit renoprotective effects and lead to the reversal of pancreatic β-cell function in streptozotocin-induced diabetes in rats. These improvements occurred via the modulation of oxidative stress, pancreatic inflammation and apoptotic pathways. Moreover, the reduced insulin injection dosage could prevent the adverse effect of insulin in prolonged treatment especially in Type 1 diabetes. Since the use of atorvastatin and insulin showed great potential benefits in the preservation of renal and pancreatic function in rats with diabetes type 1, further study is recommended to investigate whether these benefits could also be conferred in humans.

## Materials and Methods

### Animals

Male Wistar rats (200–250 g) were obtained from the National Animal Center, Salaya Campus, Mahidol University, Thailand. The animal facilities and protocols involved in the study were approved by the Laboratory Animal Care and Use Committees at the Faculty of Medicine, Chiang Mai University, Chiang Mai, Thailand (Permit No: 12/2557). All methods were performed in accordance with the relevant guidelines and regulations. All experimental rats were housed under controlled temperatures of 25 ± 1 °C and lighting in a 12 h-light/dark cycle with food and water *ad libitum*. After seven days of acclimatization, thirty-six rats were randomly divided into control (12 rats) and diabetic (24 rats) groups. The control group was divided into 2 groups, control (C), and control plus atorvastatin (CS) (six rats per group). Rats in the diabetic group were intraperitoneally (i.p.) injected with 50 mg/kg BW of streptozotocin in 10 mM citrate buffer pH 4.5 while the control rats received the equivalent dose of citrate buffer solution as a vehicle. After 7 days, rats with fasting blood glucose ≥ 250 mg/dl were included in the diabetic group and assigned into four sub-groups (six rats per group): diabetic (DM), diabetic plus insulin (DMI), diabetic plus atorvastatin (DMS), and diabetic plus insulin and atorvastatin (DMIS). Insulin (4 units/day) was injected subcutaneously while 10 mg/kg/day of atorvastatin dissolved in saline was administered orally for 4 weeks. All rats had free access to water and food and body weight was recorded daily. At the end of the experimental period, a 24-hr urine sample was collected using a metabolic cage. Rats were killed after being anesthetized using isoflurane inhalation. Blood samples were collected. Plasma and serum were separated and then stored at −20 °C until use. The kidneys were removed immediately, decapsulated and weighed to facilitate further use for the determination of renal Oat3 transporter function, malondialdehyde (MDA) concentration, hematoxylin and eosin (H&E) staining, and western blot analysis. Pancreatic tissue was collected and kept at −80 °C for further western blot analysis.

### Biochemical parameters

The plasma glucose, triglyceride, cholesterol and urine glucose levels were determined by the enzymatic colorimetric method using a commercial kit (ErbaLachemas.r.o., Brno, CZ). Plasma insulin concentration was evaluated by the Sandwich ELIZA method using a commercial kit (Rat/Mouse Insulin ELISA kit, Merck Millipore, MA, USA). Renal function was estimated by the determination of serum and urine creatinine, serum blood urea nitrogen (BUN) levels and estimated glomerular filtration rate (eGFR). Serum and urine creatinine and serum blood urea nitrogen (BUN) levels were measured using an automatic biochemical analyzer at the Clinical Laboratory, Maharaj Nakorn Chiang Mai Hospital, Chiang Mai, Thailand. eGFR was calculated using the following equation:-

1 $${\rm{eGFR}}=({\rm{urine}}\,{\rm{creatinine}}\times {\rm{urine}}\,{\rm{flow}}\,{\rm{rate}})/\mathrm{serum}\,{\rm{creatinine}}$$

### Determination of renal Oat3 function

The decapsulated kidneys were placed into freshly-oxygenated ice-cold modified Cross and Taggart saline buffer (contain: 95 mM NaCl, 80 mM mannitol, 5 mM KCl, 0.74 mM CaCl_2_, and 9.5 mM Na_2_HPO_4_, pH 7.4). Thin renal cortical slices (≤0.5 mm; 5–15 mg, wet weight) were cut using a Stadie-Riggs microtome and incubated in 1 ml of buffer containing 50 nM [^3^H] estrone sulfate (ES), a prototypical organic anion that is preferentially transported by Oat3^35,36^, to enable an uptake study for 30 mins at room temperature. At the end of the uptake period, the slices were washed in 0.1 M MgCl_2_, blotted on filter paper, weighed and dissolved in 0.4 ml of 1 M NaOH, and neutralized with 0.6 ml of 1 N HCl. Five renal cortical slices were used for each rat (5–6 rats per group). The radioactivity was measured using a liquid scintillation analyzer (Perkin Elmer, MA, USA). The transport of ES was calculated as tissue to medium (T/M) ratio.

2 $${\rm{T}}/{\rm{M}}\,{\rm{ratio}}=\mathrm{dpm}/{\rm{g}}\,{\rm{tissue}}\div\mathrm{dpm}/\mathrm{ml}\,\,{\rm{medium}}$$

### Determination of renal oxidative stress and pancreatic apoptosis

#### Determination of MDA in renal cortical tissue

The renal cortical tissue was cut and suspended in Cell Lytic MT mammalian tissue lysis/extraction reagent (Sigma Aldrich, MO, USA) containing a 1% complete protease inhibitor cocktail (Roche Applied Science, IN, USA). After being homogenized and centrifuged at 1,600 g for 10 min at 4 °C, the supernatants were collected. The MDA concentration as an indicator of renal oxidative stress condition was determined by using a commercial thiobarbituric acid reactive substance (TBARS) assay kit from Cayman Chemical (Ann Arbor, MI, USA) in line with the manufacturer’s protocol.

#### Western blot Analysis

The renal cortical or pancreatic tissues were homogenized in Cell Lytic MT mammalian tissue lysis/extraction reagent (Sigma Aldrich, MO, USA) containing a 1% complete protease inhibitor cocktail (Roche Applied Science, IN, USA) and centrifuged at 5,000 g for 10 minutes. The supernatant was collected and served as the whole cell lysate fraction and the pellet served as the nuclear fraction. The remaining supernatant was centrifuged at 100,000 g for 2 hrs, then the collected pellet served as the membrane fraction. Protein concentration was measured using a colorimetric Bradford protein assay commercially available kit (Bio-Rad, PA, USA). Total cell lysates, nuclear and membrane fractions from the renal cortex were subjected to 10% SDS-polyacrylamide gel electrophoresis (SDS-PAGE). Proteins were transferred onto PVDF membrane (Millipore, MA, USA) and were allowed to react with primary antibodies overnight at 4 °C. Antibodies against Nrf-2, IL-6, IFN-γ and PKC-α were obtained from Santa-cruz Biotechnology (CA, USA). Antibodies against GCLC and HO-1 were obtained from Abcambiochemicals (MA, USA). Antibodies against SOD2, Bcl-2, β-actin and Lamin B were obtained from Cell signaling Technology (MA, USA). Antibodies against Oat3 were obtained from Cosmo Bio Co. Ltd., (Tokyo, Japan) and antibodies against Bax, cleaved caspase-3, Na^+^-K^+^-ATPase from Merck Millipore (MA, USA). Membranes were developed using an ECL enhanced chemiluminescence agent (BioRad Laboratory Ltd., HemelHemstead, UK) and exposed using the ChemiDoc^TM^ Touch Imaging system (BioRad Laboratory Ltd., HemelHemstead, UK). Relative molecular mass of the labeled protein bands was estimated using a Page Ruler Prestained Protein Ladder (Fermentas, MA, USA), and the density was determined by the software ImageJ (National Institutes of Health, Bethesda, MD, USA). Density of the protein bands was expressed in arbitrary units relative to the respective β-actin.

### Histopathological study

To determine the changes in kidney morphology, kidneys were cut along the transverse axis then fixed in 10% neutral buffered formalin and embedded in paraffin. Paraffin-embedded specimens were cut into 2 µm-thick sections, mounted on glass slides and stained with hematoxylin and eosin (H&E) for histological assessment. The samples were observed by an observer blinded to animal treatment groups to determine the presence of glomerular and tubular changes or damage. Five slices of kidney section from each group of experiments were examined and scored under light microscope (Olympus Co., Tokyo, Japan) and evaluated the severity of renal injury score (0–4) by estimating the percentage of tubules in cortex or outer medullar and glomerulus that exhibited increases capsular space of glomerular capsule, peritubular leukocyte infiltration, tubular dilatation, and interstitial fibrosis. The histopathological evaluation was performed as follows: 0-none; 1- < 5%; 2–5–25%; 3–25–75% and 4- > 75%^37,38^.

### Statistical analysis

All data were expressed as mean ± standard error (SEM). A one-way ANOVA was used to compare the data from the various treatments followed by Fisher’s Least significant difference test (LSD). A *p* value of less than 0.05 was considered to be statistically significant.

## Electronic supplementary material

Supplementary figures
