# Supplementary material for: The additive effects of atorvastatin and insulin on renal function and renal organic anion transporter 3 function in diabetic rats
Source: Sci Rep. 2017 Oct 19;7:13532. doi: 10.1038/s41598-017-13206-5 (PMC5648883; doi:10.1038/s41598-017-13206-5)
Supplement: Supplementary file 1 — Supplementary figures [file 41598_2017_13206_MOESM1_ESM.pdf]

# The additive effects of atorvastatin and insulin on renal function and renal organic anion transporter 3 function in diabetic rats

Laongdao Thongnak[1], Anchalee Pongchaidecha[1], Krit Jaikumkao[1], Varanuj Chatsudthipong[2], Nipon Chattipakorn[1,3] and Anusorn Lungkaphin[1*]

[1]Department of Physiology, Faculty of Medicine, Chiang Mai University, Chiang Mai, Thailand

[2]Department of Physiology, Faculty of Science, Mahidol University, Bangkok, Thailand

[3]Cardiac Electrophysiology Research and Training Center, Faculty of Medicine, Chiang Mai University, Chiang Mai, Thailand

*** Corresponding author**

AnusornLungkaphin, Department of Physiology, Faculty of Medicine,

Chiang Mai University, Intravaroros Road, Chiang Mai, Thailand 50200

Tel.: 6653-945362-4; fax: 6653-945365

E-mail address: onanusorn@yahoo.com; anusorn.lungka@cmu.ac.th

Supplementary information

Figure S1 for Figure 2

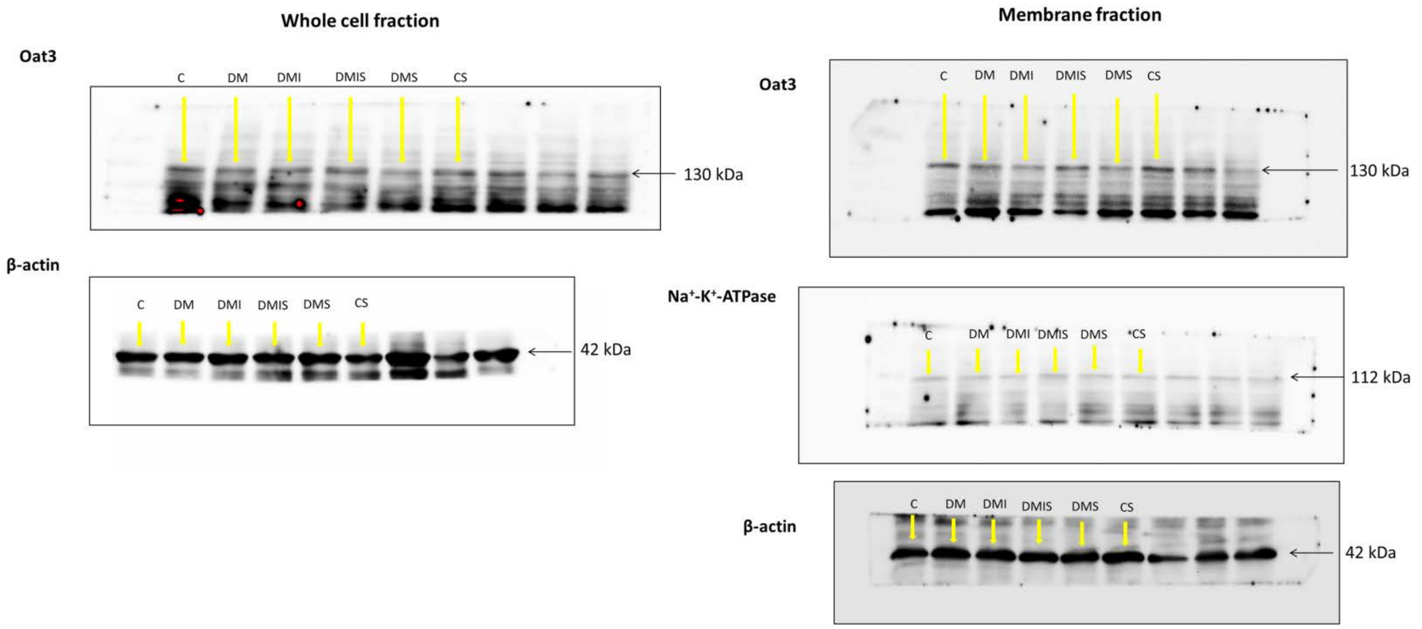

Figure S2 for Figure 3

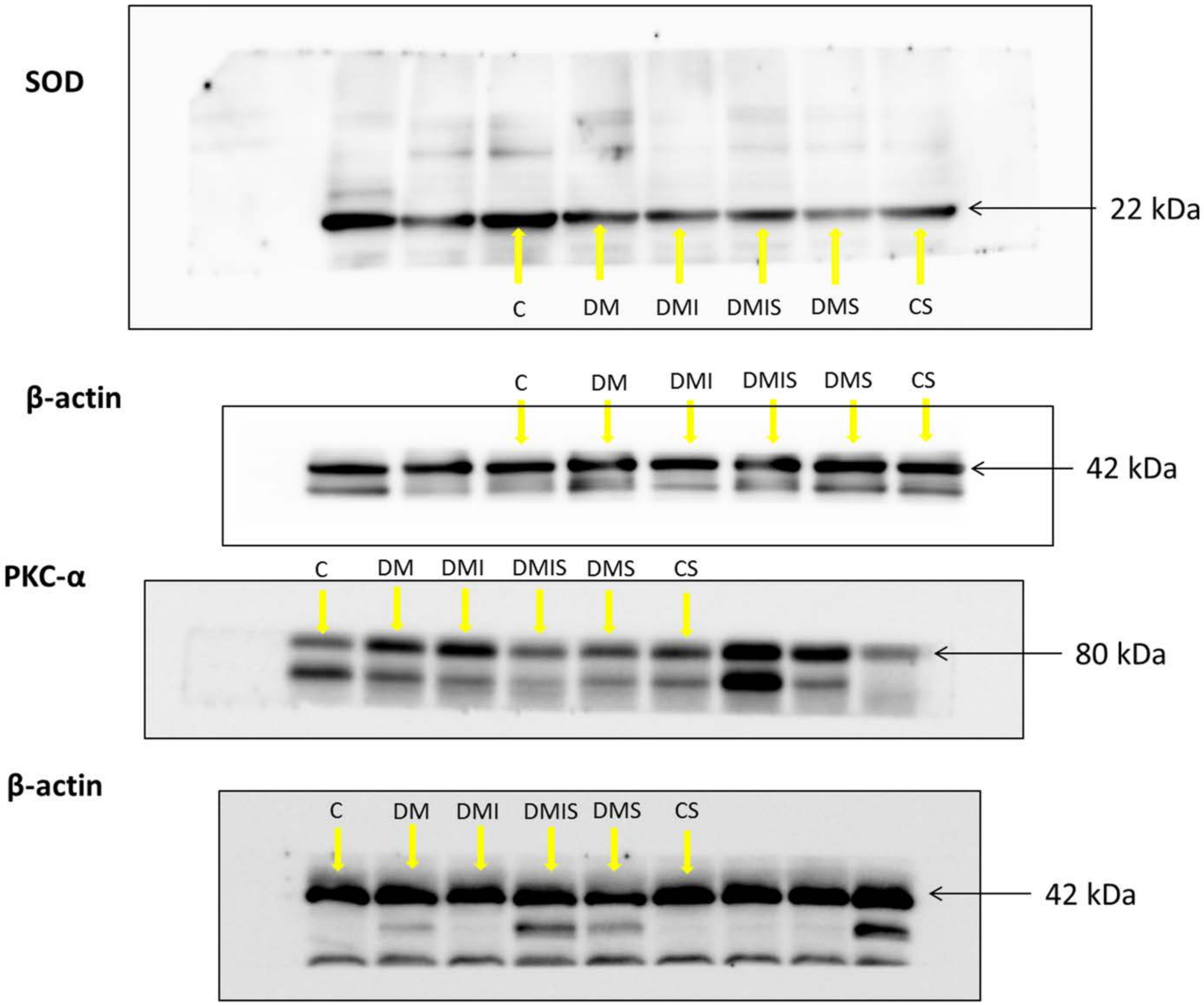

Figure S3 for Figure 4

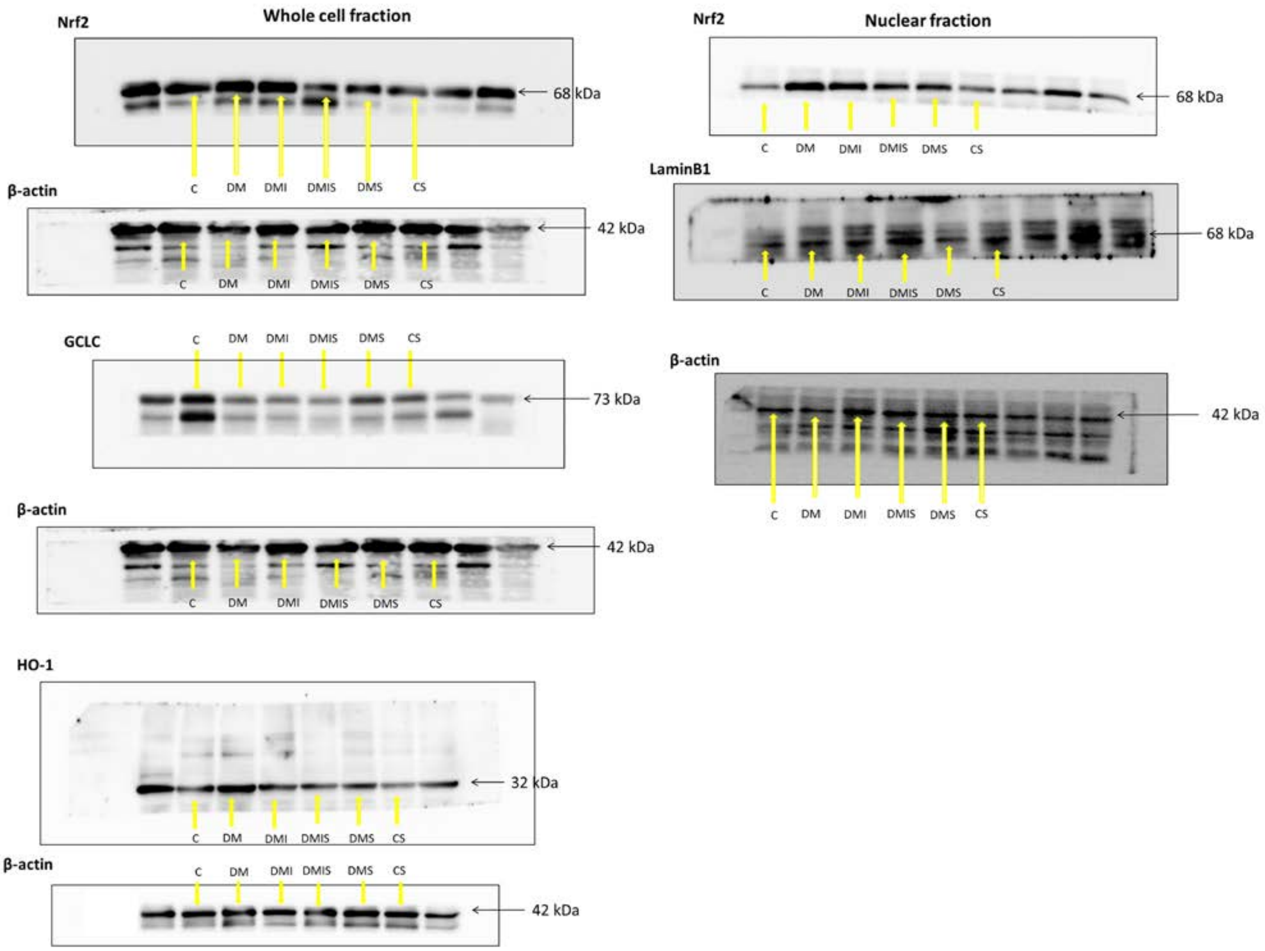

Figure S4 for Figure 5

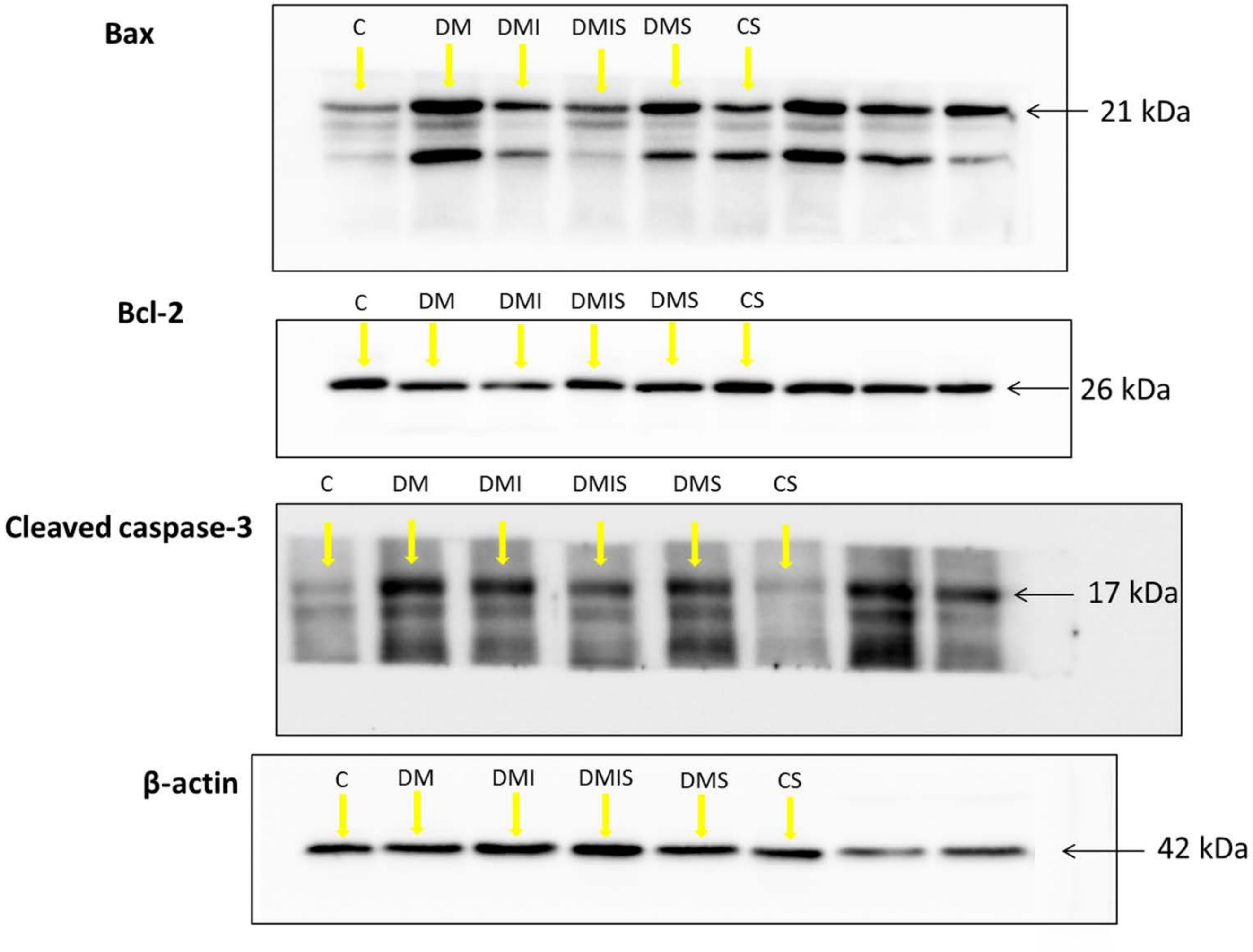

Figure S5 for Figure 6

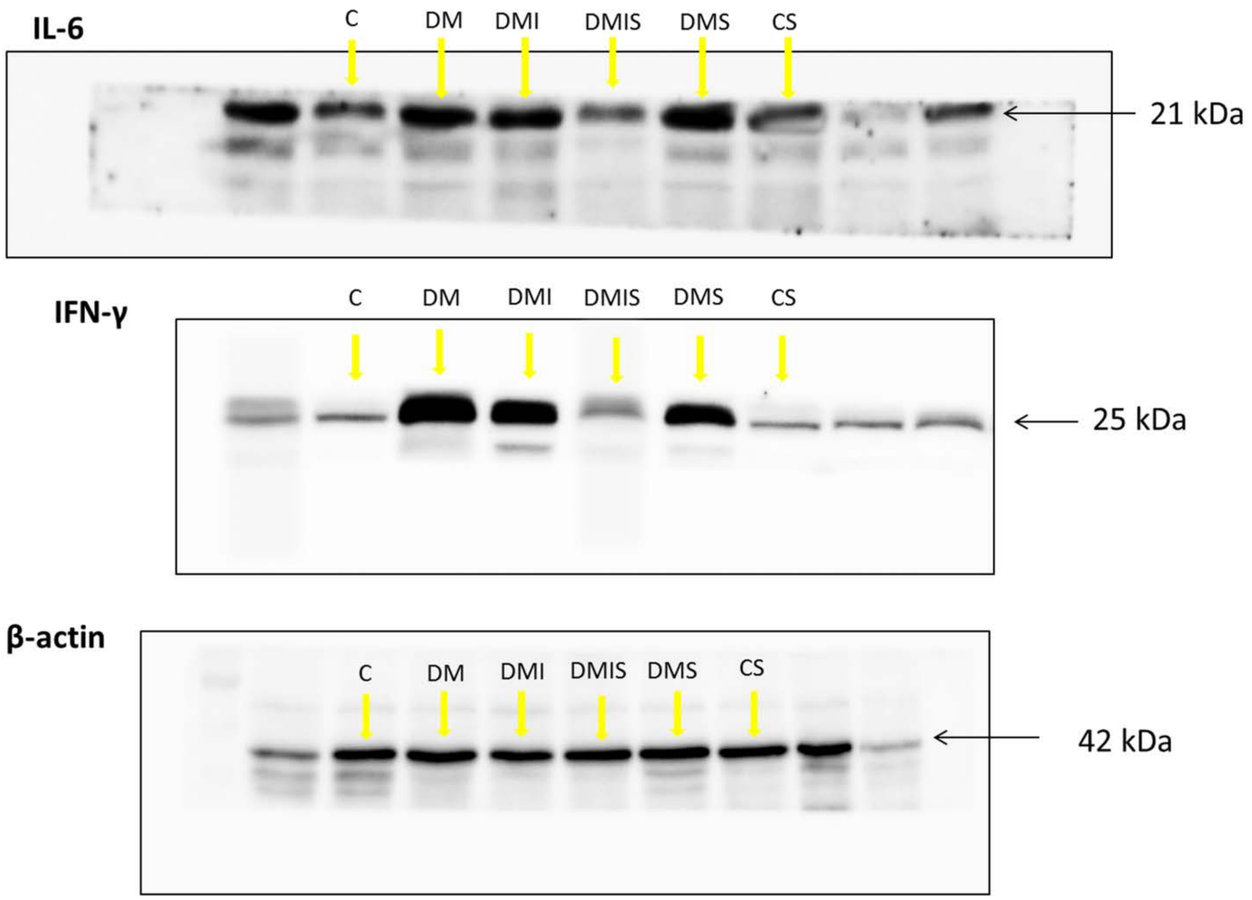

**Figure legends**

**Figure S1.** Effects of pharmacological intervention on renal cortical Oat3 expression. Western blot analysis of Oat3 expression in whole cell lysate and membrane lysate fractions (full-length blots) were shown. $Na^{+}$-$K^{+}$ ATPase was used as a marker for the membrane fraction. C - control group; DM - diabetic group; DMI - diabetic plus insulin group; DMIS - diabetic and insulin plus atorvastatin group; DMS - diabetic plus atorvastatin group; CS - control plus atorvastatin.

**Figure S2.** Effects of pharmacological intervention on renal cortical expression of SOD and PKC-α. Western blot analysis of SOD and PKC-α expression in the whole cell lysate fraction of renal cortical tissues (full-length blots) were shown. C - control group; DM - diabetic group; DMI - diabetic plus insulin group; DMIS - diabetic plus insulin and atorvastatin group; DMS - diabetic plus atorvastatin group; CS - control plus atorvastatin.

**Figure S3.** Effects of pharmacological intervention on the renal cortical expression of Nrf-2, GCLC and HO-1. Western blot analysis of Nrf2 expression in nuclear and whole cell lysate fractions, GCLC, and HO-1 of renal cortical tissues (whole cell lysate fraction) (full-length blots) were

shown. Lamin B1 was used as a marker for the nuclear fraction. C - control group; DM - diabetic group; DMI - diabetic plus insulin group; DMIS - diabetic and insulin plus atorvastatin group; DMS - diabetic plus atorvastatin group; CS - control plus atorvastatin.

**Figure S4.** Effects of pharmacological intervention on the expression of Bax, Bcl-2, and cleaved caspase-3 in pancreatic tissues. Western blot analysis of Bax, Bcl-2, and cleaved caspase-3 expressions in pancreatic tissues (full-length blots) were shown. C - control group; DM - diabetic group; DMI - diabetic plus insulin group; DMIS - diabetic and insulin plus atorvastatin group; DMS - diabetic plus atorvastatin group; CS - control plus atorvastatin.

**Figure S5**. Effects of pharmacological intervention on the expression of IL-6, and IFN-γ in pancreatic tissues. Western blot analysis of IL-6 and IFN-γ expressions in pancreatic tissues (full-length blots) were shown. C - control group; DM - diabetic group; DMI - diabetic plus insulin group; DMIS - diabetic and insulin plus atorvastatin group; DMS - diabetic plus atorvastatin group; CS - control plus atorvastatin.
